# Molecular Characterization of Carbapenem-Resistant *Acinetobacter baumannii* Isolated from Intensive Care Unit Patients in Jordanian Hospitals

**DOI:** 10.3390/antibiotics11070835

**Published:** 2022-06-21

**Authors:** Suhaila A. Al-Sheboul, Salam Z. Al-Moghrabi, Yasemin Shboul, Farah Atawneh, Ahmed H. Sharie, Laila F. Nimri

**Affiliations:** 1 Department of Medical Laboratory Sciences, Faculty of Applied Medical Sciences, Jordan University of Science and Technology, Irbid 22110, Jordan; szalmoghrabi11@ams.just.edu.jo (S.Z.A.-M.); yashboul@just.edu.jo (Y.S.); farah.hatawneh@gmail.com (F.A.); nimri01@just.edu.jo (L.F.N.); 2 Faculty of Medicine, Jordan University of Science and Technology, Irbid 22110, Jordan; ahalsharie16@med.just.edu.jo

**Keywords:** *Acinetobacter baumannii*, ICU, multidrug resistance genes, ESBL, plasmid DNA profile, Oxa carbapenems, insertion sequence, Jordan

## Abstract

*Acinetobacter baumannii* is a common cause of healthcare-associated infections (HAI) worldwide, mostly occurring in intensive care units (ICUs). Extended-spectrum beta lactamases (ESBL)-positive *A. baumannii* strains have emerged as highly resistant to most currently used antimicrobial agents, including carbapenems. The most common mechanism for carbapenem resistance in this species is β-lactamase-mediated resistance. Carbapenem-hydrolyzing class D oxacillinases are widespread among multidrug-resistant (MDR) *A. baumannii* strains. The present study was conducted to determine the presence and distribution of *bla_OXA_* genes among multidrug-resistant *A. baumannii* isolated from ICU patients and genes encoding insertion sequence (IS-1) in these isolates. Additionally, the plasmid DNA profiles of these isolates were determined. A total of 120 clinical isolates of *A. baumannii* from various ICU clinical specimens of four main Jordanian hospitals were collected. Bacterial isolate identification was confirmed by biochemical testing and antibiotic sensitivity was then assessed. PCR amplification and automated sequencing were carried out to detect the presence of *bla*_OXA-51_, *bla*_OXA-23_, *bla*_OXA-24_, and *bla*_OXA-58_ genes, and IS*Aba1* insertion sequence. Out of the 120 *A. baumannii* isolates, 95% of the isolates were resistant to three or more classes of the antibiotics tested and were identified as MDR. The most frequent resistance of the isolates was against piperacillin (96.7%), cephalosporins (97.5%), and β-lactam/β-lactamase inhibitor combinations antibiotics (95.8%). There were 24 (20%) ESBL-producing isolates. A co-existence of *bla*_OXA-51_ gene and IS*Aba1* in all the 24 ESBL-producing isolates was determined. In addition, in the 24 ESBL-producing isolates, 21 (87.5%) carried *bla*_OXA-51_ and *bla*_OXA-23_ genes, 1 (4.2%) carried *bla*_OXA-51_ and *bla*_OXA-24_, but all were negative for the *bla*_OXA-58_ gene. Plasmid DNA profile A and profile B were the most common (29%) in ESBL-positive MDR *A. baumannii* isolates while plasmid DNA profile A was the most common in the ESBL-negative isolates. In conclusion, there was an increase in prevalence of MDR-*A. baumannii* in ICU wards in Jordanian hospitals, especially those having an ESBL phenotype. Thus, identification of ESBL genes is necessary for the surveillance of their transmission in hospitals.

## 1. Introduction

*Acinetobacter baumannii* is a Gram-negative coccobacillus that is an *aerobic*, nonmotile, catalase-positive, and oxidase-negative pathogen. *A. baumannii* is an opportunistic pathogen in humans, affecting mainly people with compromised immune systems, and is becoming increasingly significant as a nosocomial infection [1]. *A. baumannii* normally inhabits mucous membranes, skin, and soil [1]. The organism is not fastidious in its growth requirement and is able to survive in both dry and moist surfaces [1]. These properties contribute to the transmission and survival in the hospital environment, which is the main reservoir for the bacterium [1]. The ability to survive in hard environmental conditions combined with its ability to accumulate the acquisition of intrinsic resistance mechanisms [2,3] leads to multidrug resistance (MDR), which intensifies the emergence and significance of this bacterium in healthcare environments. This pathogen is more common in patients hospitalized for long periods or patients having multiple invasive procedures (e.g., monitoring devices, surgical drains, mechanical ventilation, or indwelling urinary catheters), patients of advanced age, and neonates with low birth weights [1,3]. It is cultured from a hospitalized patient’s sputum or respiratory secretions, wounds, and urine, and is commonly found in irrigating solutions and intravenous fluids [4]. Furthermore, this species has been implicated in a variety of hospital-acquired infections (HAI) such as bacteremia, meningitis, and ventilator-associated pneumonia in intensive care units (ICU), as well as urinary tract infections and wound infections [1,5]. One of the most threatening characteristics of *A. baumannii* is its ability to develop resistance to carbapenems, which are broad-spectrum β-lactam antibiotics that have been used for several years as the drug of choice to treat MDR *A. baumannii* [4,6]. *A. baumannii* can acquire antibiotic-resistant genes by various mechanisms including overexpression of efflux pumps, decreased permeability, and production of antimicrobial-inactivating enzymes [4,7]. Carbapenem resistance is commonly associated with the production of two groups of β-lactamases (carbapenems); the most commonly encountered β-lactamases are carbapenem-hydrolyzing class D β-lactamases (CHDLs), and less frequently class B metallo-β-lactamases (MBLs) [8]. (CHDLs) OXA enzymes of the *Acinetobacter* spp. represent four subgroups: OXA-51-like; OXA-23-like; OXA-40-like; and OXA-58-like [1,9]. The presence of the OXA-23 subtype is considered the most common mechanism for *A. baumannii* resistance to the carbapenems [9]. OXA-51-like is intrinsic to *A. baumannii*, and its detection is a convenient method for the identification of this species [9,10]. OXA-24 shares 60% amino acid identity with OXA-23 [11]. The OXA-type carbapenems identified in *A. baumannii* include both the acquired types (OXA-23-, OXA-24/40- and OXA-58-like), where their gene clusters are either in the chromosome or plasmid [12], and the naturally occurring chromosomal OXA-51-like [13]. Studies of the genomic sequences surrounding these genes revealed the essential role of insertion sequence (IS) elements in the expression of various OXA-genes in *A. baumannii* [9,14]. The IS is a short DNA sequence that acts as a simple transposable element and that can carry resistance genes [15]. The presence of ISs such as IS*Aba-1*, IS*Aba-4*, and IS*Aba1-25*, that encode the transposases upstream of *bla*_OXA_ genes and provide promoter sequences, enhance the expression and transformation of the OXA genes [16]. Studies have revealed that IS*Aba-1*, from the IS4 family, is found upstream of *bla*_OXA-51_, *bla*_OXA-23_ and *bla*_OXA-58_ genes in *Acinetobacter* species [16]. The resistance to carbapenems mediated by *bla*_OXA-_like genes could be regulated by the upstream presence of IS*Aba-1* sequence [16]. In the current work, we present a comprehensive molecular characterization of MDR-*A. baumannii* isolates from ICU patients, which aims to determine the prevalence of ESBL isolates and their associated antibiotic resistance genes. Such an approach will help in the surveillance of MDR-*A. baumannii* among ICU patients, highlighting their prevalence, antibiotic sensitivity, and distribution.

## 2. Materials and Methods

### 2.1. Collection of A. baumannii Specimens

120 *A. baumannii* isolates were collected from patients admitted to ICUs from four Jordanian hospitals in Amman and Irbid. The 120 specimens were collected from hospitals as follows: King Abdullah University Hospital (89 isolates) in Irbid; Albasheer Hospital (7 isolates); Jordan University hospital (11 isolates); and Islamic hospitals (13 isolates) in Amman. The isolates were obtained from various clinical specimens isolated in the clinical microbiology laboratories of the mentioned hospitals. All isolates were sub-cultured on blood agar, chocolate agar, and MacConkey agar (Oxoid Ltd., Bashingstore, Hampshire, UK), and were incubated for 18–24 h at 35 °C under aerobic conditions for bacterial isolation. This study was approved by the University Review Committee for Research on Human of the King Abdulla University Hospital, and University Research Committee of the Jordan University of Science and Technology.

### 2.2. Identification of Acinetobacter baumannii Isolates

The *A. baumannii* isolates were also confirmed in this study by biochemical testing using Microgen^TM^ GnA+B-ID system (Microgen Bioproducts, Hampshire, UK). Test results were recorded based on the color change according to the color chart provided by the manufacturer. Each of the confirmed isolates was inoculated for storage into a vial containing Tryptic soy broth with 15% glycerol at −80 °C until tested. *A. baumannii* ATCC 19606 was used as a quality control strain.

### 2.3. Antibiotic Susceptibility Testing

Antibiotic susceptibility testing (AST) was performed by the Kirby–Bauer disc diffusion method using a 0.5 McFarland bacterial suspension spread over Mueller Hinton agar (Oxoid Ltd. Bashingstore Hampshire, UK). Seventeen antibiotic discs were utilized to determine the multidrug-resistant *A. baumannii* according to Clinical and Laboratory Standards Institute guidelines [17] (CLSI-2015). The susceptibility of the isolated strains was tested against amikacin (30 µg), aztreonam (30 µg), Cefepime (30 µg), Cefoperazone (75 µg), ceftazidime (30 µg), levofloxacin (5 µg) Ceftriaxone (30 µg), ciprofloxacin (5 µg), gentamycin (10 µg), imipenem (10 µg), (5 µg), meropenem (10µg), piperacillin-tazobactam (100/10 µg), piperacillin (100 µg), tetracycline (30 µg), tobramycin (10 µg), sulfamethoxazole-trimethoprim (2.75/1.25 µg), and colistin (10 µg) (all from Oxoid, UK). The strains were recorded as sensitive, intermediate, or resistant based upon CLSI-2017 guidelines for disk diffusion method–Mueller Hinton agar; *Pseudomonas aeruginosa* ATCC 27853 and *Escherichia coli* ATCC 25922 were used as quality control (QC). The QC strains for antimicrobial testing were as is recommended in the CLSI-2015 guidelines [17].

### 2.4. Identification of ESBL-Positive A. baumannii Isolates

Initial susceptibility screening of the *A. baumannii* isolates to both aztreonam (30 µg), and ceftazidime (30 µg) was evaluated by disk diffusion method, according to CLSI-2015 recommendation [17]. The double-disk diffusion method was used, and a 0.5 McFarland bacterial suspension spread over Mueller–Hinton agar (Oxoid, Hampshire, UK). A disc of ceftazidime (30 µg) and a disc of ceftazidime in combination with clavulanic acid (30/10 µg) were placed at a distance of 20 mm, incubated overnight at 37 °C and results were compared by the disk diffusion method. An increase in a zone diameter in the presence of clavulanate significantly (≥5 mm) compared to the inhibition zone around ceftazidime disc; the result is interpreted as confirmatory of ESBL production. *E. coli* ATCC 25922 and *K. pneumoniae* ATCC 700603 were used as negative and positive quality controls, respectively, according to CLSI-2015 recommendation [17].

### 2.5. Detection of bla_OXA-51_, bla_OXA-23_, bla_OXA-24_, bla_OXA-58_ Genes and the ISAba1 Insertion Sequence

Genomic DNA was extracted from 24 ESBL-positive isolated colonies using Wizard^®^ Genomic DNA Purification Kit (Promega, Madison, WI, USA). Plasmid DNA was extracted using PureYield™Plasmid Miniprep System Kit (Promega, Madison, WI, USA). DNA quantification and purity was performed using NanoDrop (ND-1000 V3.7.1) micro volume UV-Vis spectrophotometer (Thermo Scientific, Wilmington, DE, USA). The 24 ESBL-positive isolates were subjected to PCR to detect the presence of *bla*_OXA-51_*, bla*_OXA-23_*, bla*_OXA-24_*, bla*_OXA-58_ genes as described below. All primers used and PCR amplification size are listed in (Table 1) [10,11]. Each reaction mixture contained: 50–100 ng of genomic DNA, 12.5 µL PCR Master Mix, which contains Taq DNA polymerase, Qiagen PCR Buffer, and dNTPs (Qiagen, Valencia, CA, USA), and 1 µL of 20 pM of each primer, in a final volume of 25 μL. PCR condition consisted of 94 °C for 5 min, followed by 32 cycles at 94 °C for 40 s, 55 °C for 25 s, and 72 °C for 1 min, and a final extension step at 72 °C for 5 min.

The 24 ESBL-positive isolates were also assayed for the presence of the IS*Aba1* insertion sequence by PCR using the primers IS*Aba1F* and IS*Aba1R* [18]. Each reaction mixture contained: 50–100 ng of genomic DNA; 12.5 µL PCR Master Mix, which contains Taq DNA polymerase; Qiagen PCR Buffer; and dNTPs (Qiagen, Valencia, CA, USA), and 1 µL of 20 pM of each primer, in a final volume of 25 μL. The PCR conditions consisted of 95 °C for 5 min, followed by 35 cycles at 95 °C for 45 s, 56 °C for 45 s, and 72 °C for 3 min, and a final extension step at 72 °C for 5 min [18].

DNA sequencing was conducted at Princess Haya Biotechnology Center (PHBC/Jordan) for the purified PCR products of selected samples that were positive for the genes bla_OXA-23_, *bla*_OXA-24_ genes, and the insertion sequence IS*Aba1* using the primers in (Table 1). Sequencing was conducted to confirm the presence of these genes since reference QC strains were not available for all genes tested. The nested PCR is intended to reduce non-specific binding in products due to the amplification of unexpected primer binding sites. It involves two sets of primers, used in two successive PCR runs; the second set purpose was to amplify a secondary target within the first run product. The primers used to amplify a fragment in *bla*_OXA-58_ gene (Table 2) gave non-specific products. Therefore, new primers were designed to detect the *bla*_OXA-58_ gene by the same conditions of the nested PCR [17] (Table 2). Each PCR reaction mixture contained: 50–100 ng of genomic DNA, 12.5 µL Taq PCR Master Mix (Qiagen, USA), 1 µL of 20 pM of each primer, in a final volume of 25 μL. The PCR products were diluted 1:20 and 1:10 and were used as DNA template in the nested PCR. The newly designed primers used in the nested PCR are shown in (Table 2 note). PCR condition consisted of 94 °C for 5 min, followed by 32 cycles at 94 °C for 40 s, 55°C for 25 s, and 72 °C for 1 min, and a final extension step at 72 °C for 5 min. The reaction mixture contained: 50–100 ng of the diluted PCR reaction, 12.5 µL Taq PCR Master Mix (Qiagen, Valencia, CA, USA), 1 µL of 20 pM of each nested primer in a final volume of 25 μL. The second PCR run used the same amplification conditions but with the outer primers as in (Table 1 note).

### 2.6. Plasmid Profiling of MDR-A. baumannii Isolates

Plasmid DNA was extracted from 44 MDR-*A. baumannii* clinical isolates including 24 ESBL-positive isolates and 20 ESBL-negative randomly picked isolates. Extraction was performed by the centrifugation method using PureYield™ plasmid MiniPrep System (Promega, USA) and the extracted plasmid DNA was stored at −20 °C. Electrophoresis was performed on a gel containing 0.8% agarose in Tris-Boret-EDTA (TBE) buffer. Five microliters of the sample and 1μL of running dye were loaded into wells in the gel and were run for 3 h at 80 V. Gels were stained with ethidium bromide and were examined under UV illumination to visualize plasmid bands of particular size. Molecular weight of bands was estimated by comparison with molecular size markers lambda DNA hind III Digest (BioLabs, New England, UK).

### 2.7. Statistical Analysis

Statistical analysis was performed using IBM SPSS Version 21 (Armonk, NY, USA) as previously described [19,20]. A *p* value of ≤0.05 was considered statistically significant.

## 3. Results

### 3.1. Characteristics of A. baumannii Specimens

*A. baumannii* were isolated from 120 specimens as follows (Table S1, Supplementary Data): Sputum (45/120) 37.5% followed by urine (17/120) 14.2%, blood (14/120) 11.7%, wound (13/120) 10.8%, bronchial wash (12/120) 10.0%, CSF (7/120) 5.8%, triple-lumen central line (4/120) 3.3%, pus (3/120) 2.5%, peritoneal fluid (2/120) 1.7%, and (1/120) 0.8% of each tissue, ear swab, and nasal swab. Out of the 120 *A. baumannii* isolates, 57.5% (69) were collected from males and 42% (51) were from females. The hospitals where the specimens were collected from are King Abdullah University Hospital (89 isolates) in Irbid, Albasheer Hospital (7 isolates), Jordan University Hospital (11 isolates), and Islamic hospitals (13 isolates) in Amman.

### 3.2. Antibiotic Susceptibility Testing

**Figure 1:**
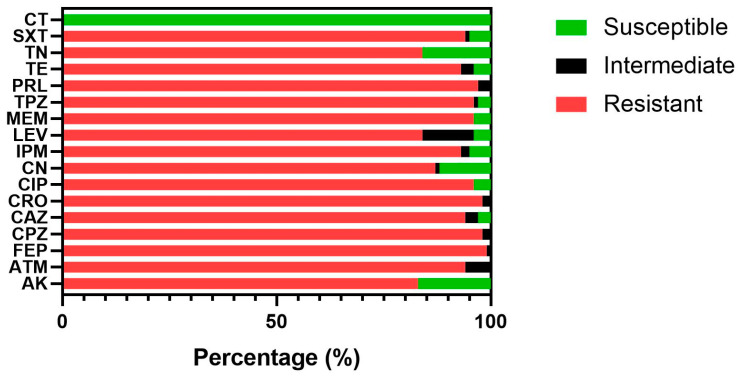
Antibiotic susceptibility testing of 120 *A. baumannii* isolates against a panel of 17 antibiotics including AK: Amikacin, ATM: Aztreonam, FEP: Cefepime, CPZ: Cefoperazone, CAZ: Ceftazidime, CRO: Ceftriaxone, CIP: Ciprofloxacin, CN: Gentamicin, IPM: Imipenem, LEV: Levofloxacin, MEM: Meropenem, TPZ: Piperacillin and Tazobactam, PRL: Piperacillin, TE: Tetracycline, TN: Tobramycin, SXT: Trimethoprim/Sulfamethoxazole, and CT: Colistin.

All 120 *A. baumannii* isolates were tested against a panel of 17 antibiotic discs as recommended by the CLSI-2015 [17]. Most of the *A. baumannii* isolates were multi-resistant to the majority of antibiotics tested (Figure 1). The highest resistance rate was observed against cefepime with 99.2% of the isolates resistant, followed by 98.3% resistance against each cefoperazone and ceftriaxone, and 96.7% for piperacillin. The resistance rate against the other antibiotics was 95.8% against each of ciprofloxacin, meropenem, and piperacillin/tazobactam; 94.2% each against aztreonam and sulfamethoxazole /trimethoprim; 93.35% against ceftazidime; 92.5% resistance against each imipenem and against tetracycline; 86.7% against gentamicin; 84.2% against levofloxacin; 83.3% against amikacin; 84.2% against tobramycin; and 0% against colistin.

### 3.3. Detection of ESBL-Positive A. baumannii Isolates

Out of the 120 tested isolates, 24 (20%) were ESBL-positive. The positive isolates were tested further by an ESBL confirmatory test. There was a statistically significant association between the ESBL production in these isolates and the resistance to 10 antibiotics (amikacin, aztreonam, ceftazidime, ciprofloxacin, gentamycin, levofloxacin, meropenem, piperacillin/tazobactam, tobramycin, and sulfamethoxazole/trimethoprim; Table 2). This association may be due to the fact that genes coding for ESBLs are carried on plasmids and these plasmids also carry resistant genes for other antibiotics. Out of the 20% (24) ESBL-positive isolates, 9.2% (11) were from sputum, 4.2% (5) from urine, 2.5% (3) from blood, and 1.7% (2) from bronchial wash, while only 0.8% (1) each was isolated from triple-lumen central line, nasal culture, and wound specimen.

### 3.4. Detection of bla_OXA-51_, bla_OXA-23_, bla_OXA-24_, bla_OXA-58_ Genes and the ISAba1 Insertion Sequence

The CHDLs OXA-type genes *bla* (OXA-58, OXA-51, OXA-24, OXA-23*)* and the insertion sequence IS*Aba1* were detected among the 24 ESBL-positive isolates at the following rates: *bla*_OXA-51_ (100%), *bla*_OXA-23_ (87.5%), *bla*_OXA-24_ (4.2%), *bla*_OXA-58_ (0%), and IS*Aba1* (100%). Our study showed significant associations (*p* < 0.05) between the bla_OXA-23_ gene and resistance to the following antibiotics: cefoperazone, ceftriaxone, ciprofloxacin, imipenem, levofloxacin, meropenem, piperacillin/tazobactam, piperacillin, and sulfamethoxazole/trimethoprim (Table 2). Additionally, statistically significant associations were identified between the OXA genes as some genes were more likely to coexist with other genes on the same chromosome or plasmid such as *bla*_OXA-23_ and *bla*_OXA-24_ (*p* < 0.05). Furthermore, the ESBL phenotype was not statistically significant with either of the OXA genes. Direct DNA sequencing was conducted for the purified PCR 

IS*Aba1* insertion sequence. The results were compared to the gene sequences at Genome BLAST, NCBI and it was a 100% match. No DNA band was observed for any of the 24 isolates in the agarose gel electrophoresis and they were considered negative for the OXA-58 gene.

### 3.5. Plasmid DNA Profiling

Plasmid profiling was conducted for 24 ESBL-positive MDR-*A. baumannii* isolates, and 20 ESBL-negative MDR-*A. baumannii* isolates to identify the number and the size of plasmids present in each. Plasmids were found in all the 24 ESBL (100%)-positive isolates that were MDR-*A. baumannii*. Additionally, plasmids were found in 18 (90%) ESBL-negative MDR- *A. baumannii* isolates, and no plasmid was found in 2 isolates (10%). The number of plasmids ranged from 1 to 10 plasmids among the ESBL-positive isolates, and 0 to 8 among ESBL-negative isolates. The size of plasmids ranged from <2 kb to 23 kb (Table S2, Supplementary Data). The plasmid profiles found in MDR-*A. baumannii* ESBL-positive and ESBL-negative isolates are shown in Table 3 and Table 4, respectively. The statistical analysis shows a significant correlation (*p* value ≤ 0.05) between plasmid number and resistance to antibiotics tested in this study (Table 5). Additionally, a significant correlation was found between the increase in plasmid number and the resistance to the following antibiotics: amikacin, cefoperazone, ceftazidime, ceftriaxone, imipenem, tetracycline, and sulfamethoxazole /trimethoprim, while no significant correlation was found between the existence of OXA genes and the number of plasmids (Table 5). The results also revealed that and all ESBL-positive MDR-*A. baumannii* isolates contain plasmids and that these isolates contain higher plasmid number than the ESBL-negative MDR-*A. baumannii* isolates. These results suggest that the genes encoding for the ESBL enzyme are carried on plasmids.

## 4. Discussion

Evolving antibiotic resistance is a clinically challenging problem among ICU patients with limited effective agents especially in cases of MDR-*A. baumannii.* [21]. In this study, 120 isolates of *A. baumannii* were isolated from various clinical specimens from patients in the intensive care units (ICU) of four main Jordanian hospitals. Of these isolates, 69 (57.5%) were from male patients, and 51 (42.5%) were from female patients with age range (<1 month to 90 years). The most common infections caused by *A. baumannii* were the respiratory tract infections (RTIs, 47.5%), followed by urinary tract infections (UTIs, 14.2%), bacteremia (11.7%), wound and pus (13.3%), cerebrospinal fluid (5.8%), triple-lumen central line (3.3%), peritoneal fluid (1.7%), ear infection, tissue infection, and nasal swab each (0.8%). The distribution of these infections varied from one hospital ICU to another. This difference might be associated with the type of procedure performed on each patient and the variations in the effectiveness of infection control programs of each hospital. A study performed in Saudi Arabia reported the most frequent infections among ICU patients is the upper respiratory tract (30.48%), followed by lower respiratory tract (47.65%) and subcutaneous tissue (9.50%) [22]. On the other hand, a study conducted in Egypt on 53 *A. baumannii* isolates from three ICUs showed that RTIs isolates counted for (45%), followed by wound infections (42%), UTIs (11%), and bacteremia (2%) [23]. In the current study, the results of the AST demonstrated a high level of resistance against most of the antibiotics tested. About 95% of the isolates were resistant to three or more classes of the antibiotics and, therefore, were identified as MDR (please check results section). The antibiotic resistance of *A. baumannii* isolates in our study was comparable to other studies conducted in the Middle East and Iran [24,25].

However, our AST showed less resistance to some of the antibiotics reported in a Chinese study which revealed that all the extensive drug-resistant *A. baumannii* isolates were resistant to all cephalosporins, all aminoglycosides, imipenem, ciprofloxacin, ampicillin/sulbactam, piperacillin/tazobactam, sulfamethoxazole-trimethoprim, and tigecycline [26]. The differences in resistance patterns observed for *A. baumannii* isolated in our study from other studies could be attributed to differences in the testing methods used, the environmental factors in each hospital, and frequent use of the antimicrobial agents. The antibiotic susceptibility test results of the QC strains, *P. aeruginosa* ATCC 27853 and *E. coli* ATCC 25922, were within the antimicrobial ranges reported in the CLSI-2015 guidelines. One exception was the inhibition zone (about 14 mm) for *P. aeruginosa* ATCC 27853 against cefepime (FEP) (30 µg), which is less than what is reported in the CLSI guidelines (24–30 mm). The explanation for this difference could be that the strain *P. aeruginosa* ATCC 27853 started to develop more resistance against cefepime, which warrants further investigation.

In our study, 24 (20%) isolates were ESBL-producing as confirmed by the ESBL confirmatory test. Our results are in accordance with another study that reported the detection of ESBL production in 27.5% of their isolates, and resistance to cefepime and ceftazidime could be detected in most of the ESBLs in Acinetobacter isolates by the double disc approximation test [27]. However, our results were much lower than what was reported in other studies that reported the presence of ESBLs in Iraq (61.53%), Iran (59%) and India (77%) of the Acinetobacter isolates [28,29,30]. The difference between these studies might be due to the method used to detect the ESBL in the isolates, where the number of ESBL-producing isolates by sulbactam was found to be much higher (77%) than the number of ESBL-producing isolates by the CLSI method (4%) [30].

Our study detected the presence of the *bla-OXA* genes in the ICU of four hospitals, which indicated that all the 24 ESBL-positive isolates (100%) were simultaneously positive for both *bla*_OXA-51_ and IS*Aba1*, whereas *bla*_OXA-51_, *bla*_OXA-23_, and IS*Aba1* were simultaneously positive in 21 (87.5%) of the tested isolates. Additionally, *bla*_OXA-51_, *bla*_OXA-24_, and IS*Aba1* were simultaneously positive in 1 (4.2%) isolate, and *bla*_OXA-51_, *bla*_OXA-23_, *bla*_OXA-24_, and IS*Aba1* were simultaneously positive in 1 (4.2%) isolate. Our findings also indicated that *bla*_OXA-23_ -like genes are predominant in *A. baumannii*, where *bla*_OXA-23_ was detected in 21 (87.5%) of the ESBL-positive isolates; our results are in agreement with a Saudi study that showed 94% of isolates carried OXA-51-like gene, and 91% contained OXA-23-like genes [31]. In contrast the *bla*_OXA-23_ gene was not dominant in Poland, and only 3 out of 104 (2.9%) carbapenem-resistant *A. baumannii* isolates carried this gene [10]. OXA-24*/OXA-40*-like carbapenemase was only detected in 1 (4.2%) isolate. OXA-58-like carbapenemase, which includes OXA-58, OXA-96 and OXA-97, was not detected in any of the *A. baumannii* isolates. The same negative results for this gene were reported by another study conducted in Poland [10] and in the UK [11]. The spread of antibiotic-resistant bacteria can be attributed to factors such as horizontal gene transfer, which refers to the transfer of genes between organisms in a way other than traditional reproduction and can even occur between different bacterial species. This horizontal gene transfer involves primarily temperate bacteriophages and plasmids [32]. Therefore, plasmid profiling was performed on 44 MDR-*A. baumannii* isolates in the current study including the 24 ESBL-positive isolates and 20 ESBL negative isolates. Results showed that all 24 ESBL-positive isolates contain plasmids with 10 plasmid profiles from A to J. Profiles A and B were the most predominant profiles present, which were found in 29% of the ESBL-positive isolates, profiles D and H were found in 8% of the ESBL-positive isolates, while profiles C, E, F, G, I, and J were found in a lower percentage (4%). Our results also showed that 18 (90%) of the 20 ESBL-negative MDR-*A. baumannii* isolates contain plasmids with profiles from A to J. Profile A was the dominant profile present in 40% of the ESBL-negative isolates; profile C was found in 10% of these isolates; and profiles B, D, E, F, G, H, I, and J were found in lower percentage (5%). The plasmid size varied in ESBL-positive isolates between ≥23,130 bp and ≥2322 to <4361 bp and were found in 35% and 20%, respectively, of the ESBL-positive isolates, while the dominant plasmid sizes in ESBL-negative isolates were longer and ranged between ≥9416 to <23,130 bp and were found in 40% of ESBL-negative isolates. The number of plasmids ranged 1 to 10 in ESBL-positive isolates and from 0 to 8 in ESBL-negative isolates. Consequently, based on the plasmid number, the MDR-*A. baumannii* isolates were divided into three groups: Group A (0–2 plasmids) was represented in 58% of the ESBL-positive isolates, and were represented in 70% of ESBL-negative isolates. Group B (3 to 4 plasmids) were represented in 12.5% ESBL-positive isolates and represented in 20% of ESBL-negative isolates. Group C (more than four plasmids) were represented in 29% of ESBL-positive isolates and represented in only 10% of the ESBL-negative isolates. All of the 44 isolates subjected to plasmid profiling were MDR-*A. baumannii*, and the results showed that 2 (10%) of the ESBL-negative isolates contained no plasmids, and 29% of the ESBL-positive isolates and 45% of the ESBL-negative isolates contained only one plasmid. These results may mean that antibiotic-resistant genes, but not ESBL genes, could be carried on the chromosome only, and that one plasmid may carry a number of genes coding for MDR. The results also revealed that all ESBL-positive MDR-*A. baumannii* isolates contain plasmids and that these isolates contain a higher plasmid number than the ESBL-negative MDR-*A. baumannii* isolates. These results also suggest that the genes encoding for the ESBL enzyme are carried on plasmids. In a study conducted in India, ten different plasmid profiles were detected among 55 *A. baumannii* clinical isolates, and most of the isolates had at least one plasmid. The number of plasmids ranged from 1 to 9, and the sizes of the plasmids ranged from 2 to >25 kb and showed that Acinetobacter may carry several copies of huge plasmids of 10 kb.

These results could be due to the presence of many transferable integron cassettes in this bacterium, which are evidently responsible for resistance [33]. A study in Iran reported seven different plasmid profiles were found in 112 Acinetobacter isolates (95.5%); the sizes of the plasmids ranged between 1 and >21 kb, and no significant association between plasmid profiles and the source of infection was found. However, they found a significant association between plasmid profiles with antibiotic resistance profiles. The study suggested that plasmid profiling is a reliable method to predict antibiotic resistance on the molecular bases [34]. MDR in *A. baumannii* is a result of acquired and innate resistance. The intrinsic resistance of *A. baumannii* is characterized by housekeeping genes encoded in the chromosomes and expressed in all strains. These genes include efflux pumps and -lactamases, such as the bla AmpC chromosomal gene, which encodes AmpC cephalosporinase, which is expressed in all strains of *Acinetobacter baumannii*. Horizontal gene transfer, a natural genetic transformation mechanism that involves the uptake of a short piece of bare DNA, allows Acinetobacter to acquire genes that encode for resistance elements [35].

## 5. Conclusions

Our results highlighted the prevalence of MDR among *A. baumannii* isolates from ICUs in four Jordanian hospitals. A significant association between the *bla*_OXA-23_ gene and resistance phenotypes was identified. Out of the 120 isolates, 24 isolates were identified as ESBL-producers. The simultaneous co-existence of insertion sequence with the naturally intrinsic *bla*_OXA-51_ gene could enhance the resistance rates to antibiotic agents. The finding that all ESBL-positive MDR-*A. baumannii* isolates contain plasmids, with higher plasmid numbers in the ESBL-positive isolates than ESBL-negative isolates, suggests that genes encoding for the ESBL enzyme are carried on plasmids. In addition, a significant association was found between the numbers of plasmids in each isolate with antibiotic resistance profiles. Our data may have an important impact on infection control policies in hospitals and on the availability of alternative treatment of such infections.

## Figures and Tables

**Table 1:** The primer sequences used to detect the presence of four *bla*_OXA_ genes and the insertion sequence IS*Aba1*.

Gene/Insertion Element	Primer	Sequence (5′–3′)	Amplicon Size (bp)	Reference
*bla*_OXA-51_^a^	F	TAA TGC TTT GAT CGG CCT TG	353	[11]
	R	TGG ATT GCA CTT CAT CTT GG		
*bla*_OXA-23_^b^	F	GAT CGG ATT GGA GAA CCAGA	501	[11]
	R	ATT TCT GAC CGC ATT TCC AT		
*bla*_OXA-24_^b^	F	GGT TAG TTG GCC CCC TTA AA	246	[11]
	R	AGT TGA GCG AAA AGG GGA TT		
*bla*_OXA-58_^b^	F	AAG TAT TGG GGC TTG TGC TG	599	[11]
	R	CCC CTC TGC GCT CTA CAT AC		
IS*Aba1* ^b^	F	CAC GAA TGC AGA AGT TG	549	[18]
	R	CGA CGA ATA CTA TGA CAC		

^a^ *A. baumannii* ATCC 19606 was used as a positive control. ^b^ No positive control strain was used, conformation of gene identity relied on finding of the expected amplified DNA band size and sequencing of the amplified fragment for the *bla*_OXA-23_, *bla*_OXA-24_ genes, and the IS*Aba1* insertion sequence. Note: *Escherichia coli* ATCC 25922 were used as negative control in all PCR reactions. New primers are designed in the present study for nested PCR to eliminate non-specific bands which were the outer primers (OXA58LF: ACGCATTTAGACCGAGCAAA and OXA58LR: CCCAGCCACTTTTAGCATA with an amplification size of 464 bp) and the inner primers (OXA58LF: TGCCAATGCACTAATTGGTTT and OXA58LR: TGCCCTTGGGCTAAATCATA with an amplification size of 315 bp).

**Table 2:** Distribution of the CHDLs OXA type genes *bla*_OXA-51,_ *bla*_OXA-23_*, bla*_OXA-24_*, bla*_OXA-58_ and the insertion sequence IS*Aba1* among 24 ESBL-positive *A. baumannii* isolates and their antibiotic suitability test results.

Antibiotics		ESBL (+)		*bla*_OXA-51_ *	*bla*_OXA-23_		*bla*_OXA-24_		*bla*_OXA-58_ *	IS*Aab1* *
		Negative	Positive	Present	Absent	Present	Absent	Present	Absent	Present
Amikacin	R	84	16	16	1	15	15	1	16	16
	S	12	8	8	2	6	8	0	8	8
	*p*-value	0.014		-	0.19		0.47		-	-
Aztreonam	I	3	4	4	1	3	4	0	4	4
	R	93	20	20	2	18	19	1	20	20
	*p*-value	0.011		N	0.408		0.648		-	-
Cefepime	I	1	0	0	0	0	0	0	0	0
	R	95	24	24	3	21	23	1	24	24
	*p*-value	0.616		-	-		-		-	-
Cefoperazone	I	1	1	1	1	0	1	0	1	1
	R	95	23	23	2	21	22	1	23	23
	*p*-value	0.285		-	0.007		0.831		-	-
Ceftazidime	I	2	2	2	1	1	2	0	2	2
	R	94	19	19	1	18	18	1	19	19
	S	0	3	3	1	2	3	0	3	3
	*p*-value	0.001		-	0.097		0.872		-	-
Ceftriaxone	I	1	1	1	1	0	1	0	1	1
	R	95	23	23	2	21	22	1	23	23
	*p*-value	0.285		-	0.007		0.831		-	-
Ciprofloxacin	R	94	21	21	1	20	20	1	21	21
	S	2	3	3	2	1	3	0	3	3
	*p*-value	0.022		-	0.002		0.699		-	-
Gentamycin	I	0	1	1	0	1	1	0	1	1
	R	86	18	18	1	17	17	1	18	18
	S	10	5	5	2	3	5	0	5	5
	*p*-value	0.046		-	0.111		0.84		-	-
Imipenem	I	2	0	0	0	0	0	0	0	0
	R	90	21	21	1	20	20	1	21	21
	S	4	3	3	2	1	3	0	3	3
	*p*-value	0.238		-	0.002		0.699		-	-

* Genes were detected in all ESBL-positive isolates. I: intermediate, R: resistant, S: scriptable.

**Table 3:** Plasmid profiles found in ESBL-positive MDR-*A. baumannii* isolates.

Plasmid Profiles	% of ESBL Isolates	Plasmid Profiling
Profile A	29	1 band ≥ 23,130 bp, (1 band ≥9416 bp–<23,130)
Profile B	29	1 band (≥9416 bp–23,13 bp)
Profile C	4	1 band ≥ 23,130 bp, (1 band ≥9416 bp–<23,130 bp), (2 bands ≥2322 bp–<4361 bp), (2 bands ≥564–<2027 bp)
Profile D	8	1 band ≥ 23,130 bp, (1 band ≥9416 bp–<23,130 bp), (1 band ≥4361 bp–<6557 bp), (1 band ≥2322 bp–<4361 bp)
Profile E	4	1 band ≥ 23,130 bp, (1 band ≥6557 bp–<9416 bp), (3 bands ≥2322–<4361 bp)
Profile F	4	2 band ≥ 23,130 bp, (3 bands ≥2322 bp–<4361 bp), (1 band ≥2027 bp–<2322 bp), (4 bands ≥564–<2027 bp)
Profile G	4	1 band ≥ 23,130 bp, (1 band ≥9416 bp–<23,130 bp), (1 band ≥4361 bp–<6557 bp)
Profile H	8	1 band ≥ 23,130 bp, (2 bands ≥9416 bp–<23,130 bp), (1 band ≥6557 bp–<9416 bp), (3 bands ≥2322 bp–<4361 bp)
Profile I	4	1 band ≥ 23,130 bp, (1 band ≥9416 bp–<23,130 bp), (3 bands ≥2322 bp–<4361 bp), (4 bands ≥564–<2027 bp)
Profile J	4	1 band ≥ 23,130 bp, (1 band ≥9416 bp–<23,130 bp), (1 band ≥6557 bp–<9416 bp), (2 bands ≥2322 bp–<4361 bp), (1 band ≥2027 bp–<2022 bp)

**Table 4:** Plasmid profiles found in ESBL-negative MDR-*A. baumannii* isolates.

Plasmid Profiles	% of ESBL Isolates	Plasmid Profiling
Profile A	40	1 band ≥9416 bp–<23,130 bp
Profile B	5	1 band ≥ 23,130 bp, (1 band ≥9416 bp–<23,130 bp)
Profile C	10	1 band ≥ 23,130 bp, (1 band ≥9416 bp–<23,130 bp), (1 band ≥4361 bp–<6557 bp), (1 band ≥567 bp–<2027 bp)
Profile D	5	1 band ≥ 23,130 bp, (1 band ≥9416 bp–<23,130 bp), (1 band ≥567 bp–<2027 bp)
Profile E	5	1 band ≥9416 bp–<23,130 bp), (1 band ≥567 bp–<2027 bp)
Profile F	5	1 band ≥ 23,130 bp, (1 band ≥9416 bp–<23,130 bp), (1 band ≥4361 bp–<6557 bp)
Profile G	5	1 band ≥ 23,130 bp, (1 band ≥9416 bp–<23,130 bp), (1 band ≥4361 bp–<6557 bp), (1 band ≥2322 bp–<4361 bp), (2 bands ≥2027–<2322 bp), (2 bands ≥567 bp–<2027 bp)
Profile H	5	1 band ≥2027–<2322 bp
Profile I	5	2 bands ≥ 23,130 bp, (1 band ≥9416 bp–<23,130 bp), (1 band ≥4361 bp–<6557 bp), (1 band ≥2322 bp–<4361 bp)
Profile J	5	(1 band ≥9416 bp–<23,130 bp), (1 band ≥567 bp–<2027 bp)

**Table 5:** Number of plasmids identified in correlation to antibiotic sensitivity testing and presence of OXA enzymes in the *Acinetobacter baumannii* isolates.

Antibiotics		Number of Plasmids									
		1	2	3	4	5	6	7	8	9	10
Amikacin	R	14	8	3	3	2	0	2	0	0	0
	S	2	2	0	1	0	2	0	1	1	1
	*p*-value	0.022									
Aztreonam	I	2	0	0	0	0	1	1	0	0	0
	R	14	10	3	4	2	1	1	1	1	1
	*p*-value	0.344									
Cefepime	R	16	10	3	4	2	2	2	1	1	1
	*p*-value	-									
Cefoperazone	I	0	0	0	0	0	1	0	0	0	0
	R	16	10	3	4	2	1	2	1	1	1
	*p*-value	0.015									
Ceftazidime	I	0	1	0	0	0	0	0	1	0	1
	R	15	9	3	4	2	1	2	0	0	0
	S	1	0	0	0	0	1	0	0	1	0
	*p*-value	<0.001									
Ceftriaxone	I	0	0	0	0	0	1	0	0	0	0
	R	16	10	3	4	2	1	2	1	1	1
	*p*-value	0.015									
Ciprofloxacin	R	15	9	3	4	2	1	2	1	1	1
	S	1	1	0	0	0	1	0	0	0	0
	*p*-value	0.662									
Gentamicin	I	0	0	0	0	0	1	0	0	0	0
	R	15	8	3	3	2	0	1	1	1	1
	S	1	2	0	1	0	1	1	0	0	0
	*p*-value	0.056									
Imipenem	I	0	0	0	0	0	0	0	1	0	0
	R	15	9	3	4	2	1	2	0	1	1
	S	1	1	0	0	0	1	0	0	0	0
	*p*-value	<0.001									
Levofloxacin	I	5	1	0	0	0	0	0	0	0	0
	R	10	8	3	4	2	1	2	1	1	1
	S	1	1	0	0	0	1	0	0	0	0
	*p*-value	0.763									
Meropenem	R	15	9	3	4	2	1	2	1	1	1
	S	1	1	0	0	0	1	0	0	0	0
	*p*-value	0.662									
Piperacillin/Tazobactam	I	0	1	0	0	0	0	0	0	0	0
	R	15	9	3	4	2	1	2	1	1	1
	S	1	0	0	0	0	1	0	0	0	0
	*p*-value	0.761									
Piperacillin	I	0	0	0	0	0	1	0	0	0	0
	R	15	10	3	4	2	1	2	1	1	1
	S	1	0	0	0	0	0	0	0	0	0
	*p*-value	0.227									
Tetracycline	I	0	0	0	0	0	0	0	0	1	0
	R	15	10	2	4	2	2	2	0	0	1
	S	1	0	1	0	0	0	0	1	0	0
	*p*-value	<0.001									
Tobramycin	R	14	8	3	3	2	0	1	1	1	1
	S	2	2	0	1	0	2	1	0	0	0
	*p*-value	0.204									
Trimethoprim/Sulfamethoxazole	R	16	9	3	4	2	1	2	0	0	0
	S	0	1	0	0	0	1	0	1	1	1
	*p*-value	0.001									
Colistin	S	16	10	3	4	2	2	2	1	1	1
	*p*-value	-									
ESBL	(−)	9	3	2	2	1	0	0	1	0	0
	(+)	7	7	1	2	1	2	2	0	1	1
	*p*-value	0.485									
*bla*_OXA-51_	(+)	7	7	1	2	1	2	2	0	1	1
	*p*-value	-									
*bla*_OXA-23_	(−)	1	1	0	0	0	1	0	0	0	0
	(+)	6	6	1	2	1	1	2	0	1	1
	*p*-value	0.879									
*bla*_OXA-24_	(−)	6	7	1	2	1	2	2	0	1	1
	(+)	1	0	0	0	0	0	0	0	0	0
	*p*-value	0.962									
*bla*_OXA-24_	(−)	7	7	1	2	1	2	2	0	1	1
	*p*-value	-									

I: intermediate, R: resistant, S: scriptable.

## Data Availability

Data is contained within the article or supplementary material and also available upon request.

## Supplementary Materials

The following supporting information can be downloaded at: https://www.mdpi.com/article/10.3390/antibiotics11070835/s1, Table S1: Type and number of samples, and their percent out of the 120 collected. Table S2. Size of plasmids present in the MDR-*A. baumannii* ESBL-positive, and ESBL-negative isolates.
