# Supplementary material for: Molecular Characterization of Carbapenem-Resistant Acinetobacter baumannii Isolated from Intensive Care Unit Patients in Jordanian Hospitals
Source: Antibiotics (Basel). 2022 Jun 21;11(7):835. doi: 10.3390/antibiotics11070835 (PMC9311868; doi:10.3390/antibiotics11070835)
Supplement: Supplementary file 1 [file antibiotics-11-00835-s001.zip › antibiotics-1779197-supplementary.pdf]

Supplementary data

Molecular Characterization of Carbapenem-Resistant *Acineto-bacter baumannii* Isolated from Intensive Care Unit Patients in Jordanian Hospitals

Suhaila A. Al-Sheboul [1,*], Salam Z. Al-Moghrabi [1], Yasemin Shboul [1], Farah Atawneh [1], Ahmed H. Sharie [2] and Laila F. Nimri [1]

Table S1: Type and number of samples, and their percent out of the 120 collected.

Sample source	Number (Percentage %)
Sputum	45 (37.5)
Urine	17 (14.2)
Blood	14 (11.7)
Wound	13 (10.8)
Bronchial wash	12 (10.0)
Cerebrospinal spinal fluid	7 (5.8)
Triple-lumen central line	4 (3.3)
Pus	3 (2.5)
Peritoneal fluid	2 (1.7)
Ear swap	1 (0.8)
Tissue (unidentified from the source)	1 (0.8)
Nasal swab	1 (0.8)

Table S2. Size of plasmids present in the MDR- *A.baumannii* ESBL- positive, and ESBL-negative isolates.

**Size of plasmid (bp)**	**ESBL -Positive isolates**		**ESBL- negative isolates**	
	**Number**	**Percentage (%)**	**Number**	**Percentage (%)**
≥23,130	29	35	8	19
≥9,416-<23,130	14	17	17	40
≥6,557-<9,416	4	5	0	0
≥4,361-<6,557	6	8	5	12
≥2,322-<4,361	17	20	2	5
≥2,027-2,322	3	4	2	5
≥564-<2,027	10	12	8	19
